# Commentary: Vasodilator Myocardial Perfusion Cardiac Magnetic Resonance Imaging Is Superior to Dobutamine Stress Echocardiography in the Detection of Relevant Coronary Artery Stenosis: A Systematic Review and Meta-Analysis on Their Diagnostic Accuracy

**DOI:** 10.3389/fcvm.2021.694323

**Published:** 2021-06-10

**Authors:** Attila Kardos, Roxy Senior, Harald Becher

**Affiliations:** ^1^Department of Cardiology, Milton Keynes University Hospital, Milton Keynes, United Kingdom; ^2^School of Sciences and Medicine, University of Buckingham, Buckingham, United Kingdom; ^3^Imperial College, National Heart and Lung Institute, London, United Kingdom; ^4^ABACUS, Mazankowski Alberta Heart Institute, University of Alberta Hospital, Edmonton, AB, Canada

**Keywords:** stress echocardiography, cardiac MRI, chronic coronary syndrome, diagnostic accuracy and yield, discussion points

We are grateful to the authors for sharing the results of this very precise and detailed analysis of comparing the diagnostic performance of perfusion cardiac magnetic resonance (pCMR) and dobutamine stress echocardiography (DSE) for the detection of coronary artery stenosis with the scientific readership as the two functional test modalities without associated harmful radiation (1). The authors found higher sensitivity for pCMR vs. DSE (0.88 vs. 0.720) with a negative likelihood ratio of 0.14 vs. 0.31, respectively. There was no difference in specificity. We acknowledge the precise nature of the work. However, we would like to raise some points that may be worthwhile considering.

(1) This meta-analysis takes historical studies into account using either DSE or pCMR that compared the functional test results to that of invasive or coronary CT angiography (CCTA) or invasive fractional flow reserve. Albeit these are the only data available for comparison, it may question the legitimacy of comparing two functional tests with different principles to address coronary artery disease (CAD) severity detection. With this in mind, one would look for studies that are comparing the effect of the same stressor (e.g., coronary vasodilators) that investigates the accuracy of the imaging modality, i.e., echocardiography vs. CMR in detecting inducible ischemia and significant CAD. One such methodological comparison was showing no difference in the accuracy between echocardiography vs. CMR using vasodilator stress test in the same patients' cohort (2).

(2) The majority of the included DSE studies were performed before 2000 without using ultrasound-enhancing contrast agents (UECAs). Not until 2009, the European Association of Cardiovascular Imaging recommended UECA to be used regularly during echocardiography where >2 segments of the left ventricle are not delineated properly (3). This certainly was a major step forward in improving interpretability and increasing operator confidence during stress echocardiography. However, recent comparisons of contrast-enhanced stress echocardiography with coronary angiography mainly used vasodilator stress test. Further randomized, prospective studies with contemporary imaging techniques and modalities, e.g., contrast-enhanced stress echocardiography, may help our understanding of the strength and weaknesses of those modalities.

(3) Although the diagnostic accuracy is essential, the prediction of outcome and/or risk stratification following a test is probably more important. In this respect, both pCMR (4–6) and DSE (7–9) have robust data, although with no head-to-head comparative studies. Stress echocardiography has consistently shown that a normal study identifies a low-risk cohort who needs no further testing, while significant ischemia identifies a high-risk group. In addition, the Mayo Clinic group has shown the same outcome in patients with abnormal stress echo findings regardless of the degree of coronary artery stenosis by Invasive Coronary Angiography (10). This meta-analysis did not evaluate outcome prediction nor risk stratification.

(4) Thus, current European Society of Cardiology and American Heart Association guidelines for chest pain assessment in chronic coronary syndrome patients with intermediate pretest probability recommend a non-invasive functional test [stress echocardiography, single-photon emission computed tomography, CMR] as well as an anatomical test, such as CCTA as the initial test, guided by the local expertise and infrastructure (11). In order to recommend CMR as a first-line diagnostic test, further comparative studies on risk stratification, management-based outcome, and cost-effectiveness need to be demonstrated.

## Author Contributions

All authors listed have made a substantial, direct and intellectual contribution to the work, and approved it for publication.

## Conflict of Interest

The authors declare that the research was conducted in the absence of any commercial or financial relationships that could be construed as a potential conflict of interest.
